# LC–ESI–IT-MS/MS and MALDI-TOF Approach: Identification of Natural Polymers from *Rhizophora mangle* Barks and Determination of Their Analgesic and Anti-inflammatory Properties

**DOI:** 10.1007/s13659-018-0192-8

**Published:** 2018-11-14

**Authors:** Leonardo Mendes de Souza Mesquita, Caroline Fabri Bittencourt Rodrigues, Cláudia Quintino da Rocha, Mayara Silveira Bianchim, Clenilson Martins Rodrigues, Vanda Maria de Oliveira, Henrique Hessel Gaeta, Mariana Novo Belchor, Marcos Hikari Toyama, Wagner Vilegas

**Affiliations:** 1 Laboratory of Bioprospection of Natural Products (LBPN), UNESP - São Paulo State University/Coastal Campus of São Vicente, Pça Infante Dom Henrique S/N, São Vicente, São Paulo CEP: 11330-900 Brazil; 2 Laboratório de Estudos Avançados em Fitomedicamentos (LEAF), UFMA - Federal University of Maranhão, Av. dos Portugueses, 1966 - Bacanga, São Luís, Maranhão CEP: 65080-805 Brazil; 3 Embrapa Agroenergy, Brazilian Agricultural Research Corporation, W3 Norte, PqEB, Brasília, DF 70770-901 Brazil

**Keywords:** Catechins, Tannins, Morphine-like effect, Anti-inflammatory, Mangroves

## Abstract

**Abstract:**

We recognize the chemical composition of the acetonic extract of *Rhizophora mangle* barks (AERM) using mass spectrometry analysis [liquid chromatography (LC)–ESI–IT-MS/MS and matrix-assisted laser desorption/ionization-time of flight-MS (MALDI-TOF)]. Analgesic activity was evaluated by formalin and tail-flick experimental assays. Anti-inflammatory activity was performed by paw edema test induced by carrageenan and 48/80 compounds. The first series of experiments involved [LC]–FIA–IT-MS/MS with 11 separated catechins derivatives until degree of polymerization 3 (DP3). The spectra obtained by MALDI-TOF analysis of the AERM presented two homologous series: one based on polymers of *m*/*z* 288 Da increments (up to DP12) and another series based on polymers of *m*/*z* [288 + 162] Da increments (up to DP11). In addition to these series of flavan-3-ol, each DP had a subset of masses with a variation of − 16 Da (homologous series of afzelechins—*m*/*z* 873–3465 Da) and + 16 Da (homologous series of gallocatechins—*m*/*z* 905–3497 Da). A similar pattern with homologous series of gallocatechins and afzelechins could also be observed for a fifth and a sixth monohexoside series: glucogallocatechins (*m*/*z* 779–3371) and glucoafzelechins (*m*/*z* 747–3339). The intraperitoneal administration of different doses of AERM (50, 150 and 300 µg mL^−1^) have a morphine-like effect and intense anti-inflammatory activity.

**Graphical Abstract:**

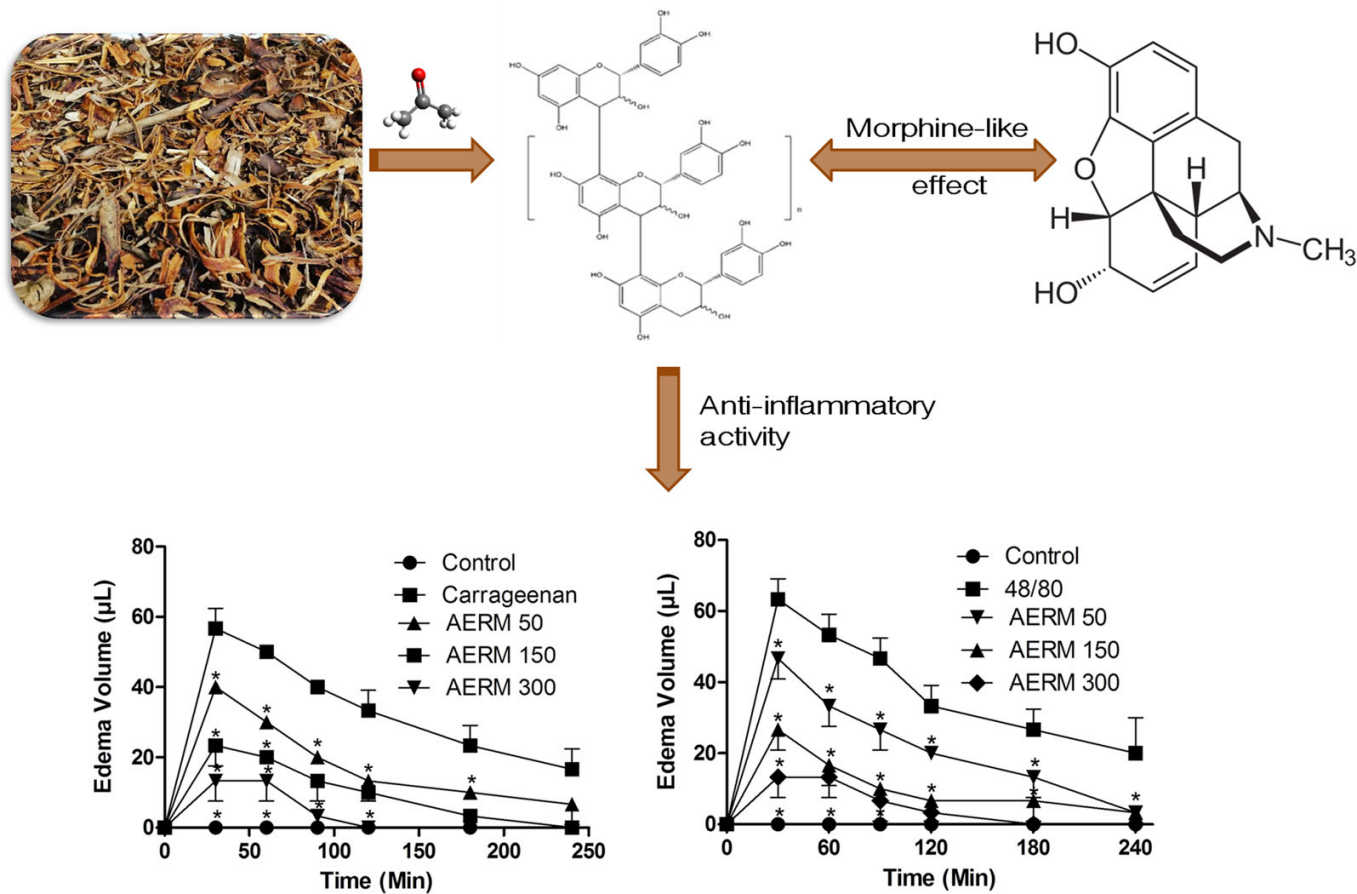

## Introduction

*Rhizophora mangle* L. (Rhizophoraceae) is a pioneer plant that dwelleth the estuarine ecosystem. Mangrove plants are potential sources of biologically active compounds with wide application in ethnopharmacology practices. These species dwell in stressful environmental conditions (salinity, tidal fluctuations, and anoxic soil), and are adapted to this harsh ecosystem having many substances that protect them from these conditions [1].

In traditional medicine, *R. mangle* is commonly used to treat diarrhea, ulcers, inflammation, tumors, diabetes, and pain [1, 2]. Pain is the major reason for seeking medical consultations and represents high medical and economic costs to the community [3]. Opioids are the most used resource to treat this condition, but the continuous use of this compound can generate dependence and side effects, such as gastrointestinal problems and respiratory depression. Therefore, the search for new analgesics, especially those of natural origin, is a need and a challenge for medicinal chemistry [4].

Inflammatory diseases, despite being described many years ago, have no fully effective treatment, there are many side reactions caused by anti-inflammatory drugs. Therefore, a significant commercial interest to discover new anti-inflammatory drugs with fewer side effects has been observed nowadays [5]. Corticosteroids and nonsteroidal anti-inflammatories remain the major therapeutic resources for inflammation control [6], but these drugs induce significant side effects, especially when used for a prolonged period, as overload kidneys and liver, osteoporosis and anaphylactic shock [7].

The polyphenolic compounds such as proanthocyanidins (PAs-tannins) consisting of oligomers and polymers by flavan-3-ol units the are most abundant in *Rhizophora* [8]. Due to its variable degree of polymerization (DP), the isolation and identification of the PAs require the use of complex steps of separation and purification. Additionally, PAs are highly unstable compounds with complex molecular structures, thermolabile and photosensitive. Therefore, mass spectrometry (MS) techniques based on matrix-assisted laser desorption/ionization-time of flight-MS (MALDI-TOF-MS) and liquid chromatography coupled to the tandem MS (LC–MS) has been attracting attention because of their ability to reliable analysis. In this context, the purpose of the present study was to characterize the chemical composition of the acetone extract of *R. mangle* bark’s (AERM) and evaluate the analgesic and anti-inflammatory activity, which can be an excellent alternative for the development of new drugs.

## Results and Discussion

Previous studies carried out with species of the genus *Rhizophora* and Rhizophoraceae family have demonstrated that these specimens are important sources of polyphenolic compounds (tannins) [2, 8]. MS experiments were performed using LC–MS system equipped with an ESI source and an Ion Trap analyser was made to investigate the presence of different compounds with the same molecular weight and then to perform a qualitative analysis on the PAs constituents occurring in the AERM.

**Fig. 1:**
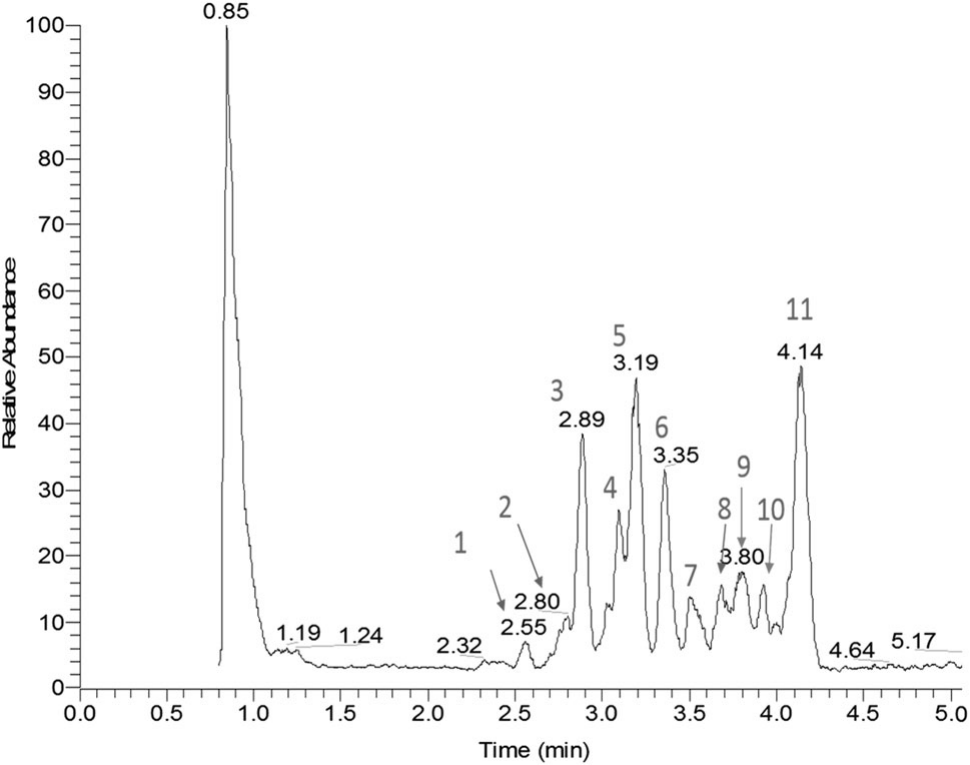
Total ion chromatogram in negative ion UPLC-MS analysis of proanthocyanidins presents in the AERM

In order to recognize the main class of compounds, we performed LC–ESI–MS/MS analysis. The chromatographic separation was optimized to obtain an excellent separation. The LC–ESI–MS/MS obtained has satisfactory chromatographic resolution (Fig. 1), with 11 separate compounds and their structure proposed in Table 1.

**Table 1:** LC–[MS/MS] fragmentations performed during the chromatographic analysis of the condensed tannins from AERM in the negative ionisation mode

Peaks	Tr (min)	[M−H]^−^ (*m*/*z*)	MS^n^ events	Proposed IDs
**1**	2.55	577	MS^2^: 425 MS^3^: 407, 272, 298, 228	Catechin dimer
**2**	2.80	723	MS^2^: 571, 577, 269	Catechin dimer + deoxyhexose
**3**	2.89	577	MS^2^: 425	Catechin dimer
**4**	3.10	435	MS^2^: 289, 136	Catechin + deoxyhexose
**5**	3.19	739	MS^2^: 577 MS^3^: 559, 451, 289, 269	Catechin dimer + hexose
**6**	3.35	451	MS^2^: 341, 289	Catechin + hexose
**7**	3.52	577	MS^2^: 425	Catechin dimer
**8**	3.60	577	MS^2^: 425	Catechin dimer
**9**	3.80	451	MS^2^: 341	Catechin + hexose
**10**	3.95	451	MS^2^: 341	Catechin + hexose
**11**	4.14	451	MS^2^: 341	Catechin + hexose

Compounds **1**, **3**, **7** and **8** were identified as catechin (CA) dimers based on their [M−H]^−^ precursor ion at *m*/*z* 577, as well as after MS^2^ fragmentation, which resulted in the products ions at *m*/*z* 425, *m*/*z* 407, *m*/*z* 272, *m*/*z* 298 and *m*/*z* 228, consistent with the data reported in the literature for CA dimmers [9]. Compound **2** (*m*/*z* 723, [M−H]^−^) was assigned as a CA dimer with one deoxyhexose moiety; MS^2^ fragmentation of this precursor ion led to the products ions at *m*/*z* 577, *m*/*z* 571 and *m*/*z* 269 [10]. Fragmentation of the precursor ion of *m/z* 435 [M−H]^−^ (compound **4**) led to the product ions at *m*/*z* 289 [M−146−H]^−^ and *m*/*z* 136, thus suggesting a CA core bounded to a deoxyhexose unit. Fragmentation of the compound **5** (*m*/*z* 739, [M−H]^−^) led to the fragments at *m*/*z* 577 [M−162−H]^−^, *m*/*z* 559, *m*/*z* 451, *m*/*z* 289 and *m*/*z* 269 [10], corresponding to a CA core bounded to a hexose moiety. Compounds **6**, **9**, **10** and **11** were also identified as hexosyl-catechins, with *m*/*z* 451 [M−H]^−^ and MS^2^ product ions at *m*/*z* 341 [M−162−H]^−^.

There are two isomers of CAs: (+)-CA, and (−)-epicatechin (EC), they are considered geometric isomers, where CA in a *trans* form and EC in a *cis* form [11]. Their structure consists of two benzene rings (A and B rings) connected through a pyran ring C. The subtle difference in molecular structure may result in drastic differences in pharmacological processes and therapeutic efficacy [12]. Besides we cannot confirm the presence of these isomers by LC–MS/MS analysis, we suggest, that AERM possess these compounds. We detected for the compounds **1**, **3**, **7**, **8** and compounds **6**, **9**, **10** and **11** different retention times and same *m*/*z*, thus strongly suggesting the presence of isomers.

CAs are classified as flavan-3-ol monomers and have the capacity to polymerize, forming oligomeric compounds with high molecular weight [13]. The polymeric character is evidenced by the chromatogram; however, the characterization of condensed tannins was limited with ESI–IT-MS/MS experiments. To solve this bottleneck, analysis using MALDI-TOF MS was performed to establish the DP to the PAs and its glucosylated forms.

**Fig. 2:**
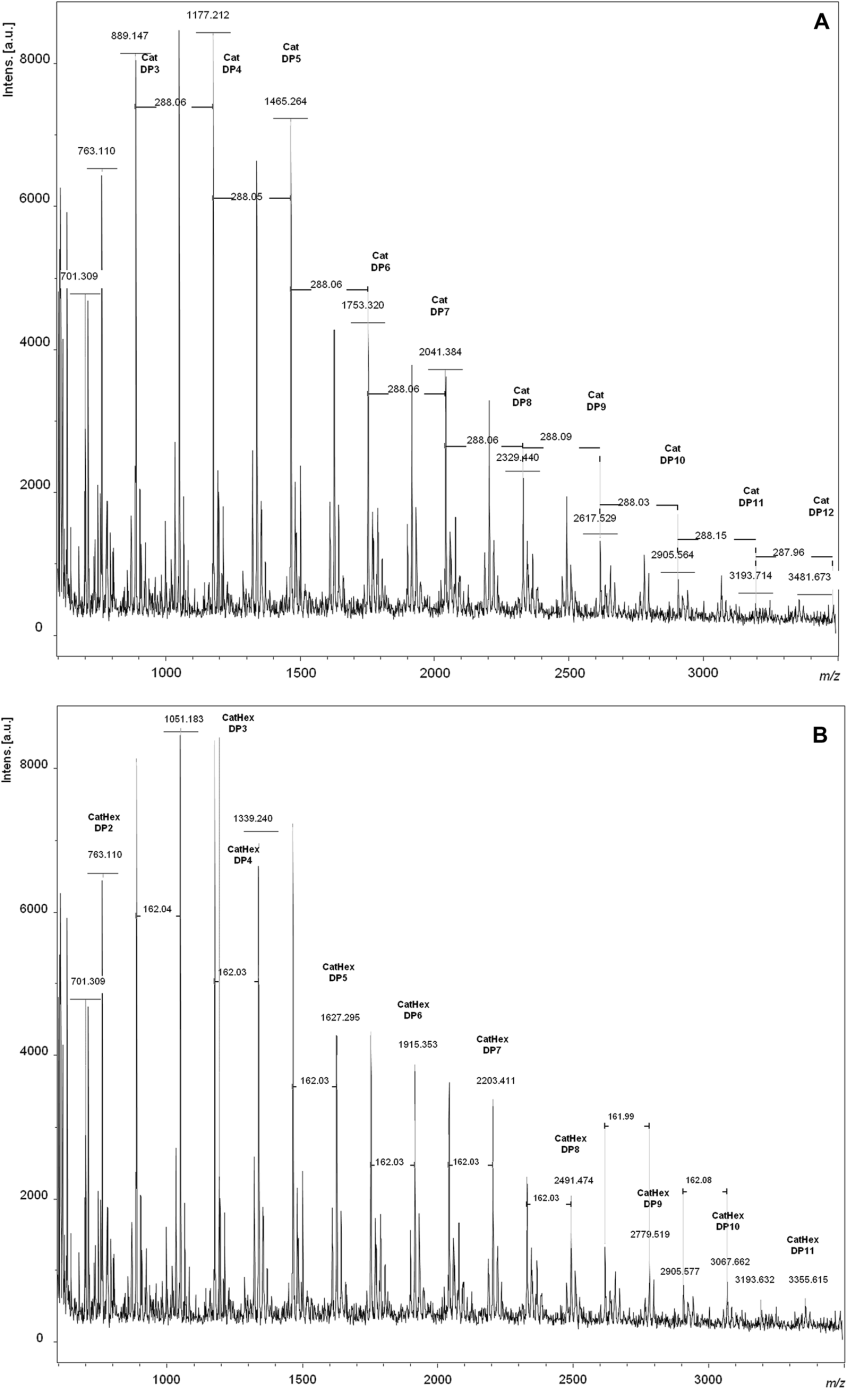
MALDI-TOF positive reflectron mode mass spectra of the condensed tannins from *R. mangle*. **A** catechins series with varying hydroxylation patterns, **B** catechins series with hexose group and varying hydroxylation patterns

The spectra obtained by MALDI-TOF analyzes of the AERM presented two main homologous series: one with 288 Da increments and another one with 288 + 162 Da increments, corroborating with those found using LC–ESI–IT-MS/MS technique. The series containing 288 Da increments were attributed to the polymeric CAs, according to formula 290 + 288 (n − 1) + 23 (n = DP; + 23 Da was attributed due to sodium adducts). Sodium adducts with up to DP12 (3481.673 Da) were found, even with the decrease in the intensity of the intercepted ions (Fig. 2a). In addition to this first series of flavan-3-ol, each DP had a subset of masses with a variation of − 16 and + 16 Da (Fig. 2a). These subsets can be explained by heteropolymers of repeating flavan-3-ol units containing an additional hydroxyl group (∆ + 16 Da), characterizing a homologous series of gallocatechins (*m*/*z* 905.145–3497 Da) (Fig. 2a). The other series consists in heteropolymers of repeating flavan-3-ol units without a hydroxyl group (∆ − 16 Da), that is, a homologous series of afzelechins (*m*/*z* 873–3465 Da) (Fig. 2a).

MALDI-TOF MS experiments indicated the occurrence of a mixture of PAs and its monohexoside derivatives, which presented a variable DP (from 2 up to 12). Each precursor ion of the condensed tannins from AERM was always followed by mass signals at 162 Da, suggesting the presence of a series of monohexoside anthocyanidins, that can be explained by the formula 290 + 288 (n − 1) + 162 + 23 (Fig. 2b). Condensed tannins with a hexose group with DP2–11 (*m*/*z* 763.110–3355.615) were detected in AERM (Fig. 2b). Species with up to DP11 CatHex (*m*/*z* 763.110–3355.615 Da) could be observed (Fig. 2b).

The same pattern with homologous series of gallocatechins and afzelechins could also be observed for monohexoside series, thus evidencing that the extract also possesses a series of monohexoside gallocatechins (*m*/*z* 779–3371) as well as a series of monohexoside afzelechins (*m*/*z* 747–3339). The peaks with the highest intensities in MALDI-TOF MS of the AERM were summarized in Table 2.

**Table 2:** Summary of peaks with the highest intensities in MALDI-TOF MS of the condensed tannins from AERM

Polymers	N1 catechins	N2 afzelechins	N3 gallocatechins	N4 hexoside	MW + Na
DP2	2	0	0	0	763.110
DP3	3	0	0	0	889.147
	3	0	0	1	1051.18
	2	1	0	0	747.133
	2	0	1	0	905.145
	1	2	0	0	–
	1	0	2	0	–
DP4	4	0	0	0	1177.212
	4	0	0	1	1339.240
	4	1	0	0	–
	3	0	1	0	905.145
	3	1	0	1	1035.211
	3	0	1	1	1067.178
	2	2	0	0	–
	2	0	2	0	
	2	2	0	1	
	2	0	2	1	
	1	3	0	0	
	1	0	3	0	
DP5	5	0	0	0	1465.264
	5	0	0	1	1627.295
	4	1	0	0	–
	4	0	1	0	1193.210
	4	1	0	1	1323.266
	4	0	1	1	1355.230
	3	2	0	0	–
	3	0	2	0	–
	3	2	0	1	–
	3	0	2	1	1084.189
	2	3	0	0	–
	2	0	3	0	–
	1	4	0	0	–
	1	0	4	0	–
DP6	6	0	0	0	1753.320
	6	0	0	1	1915.353
	5	1	0	0	–
	5	0	1	0	1481.252
	5	1	0	1	1611.313
	5	0	1	1	1643.291
	4	2	0	0	–
	4	0	2	0	–
	4	2	0	1	–
	4	0	2	1	1372.240
	3	3	0	0	–
	3	0	3	0	–
	2	4	0	0	1699.293
	2	0	4	0	–
	1	5	0	0	–
	1	0	5	0	–
DP7	7	0	0	0	2041.384
	7	0	0	1	1769.318
	6	1	0	0	1738.328
	6	0	1	0	–
	5	2	0	0	–
	5	0	2	0	2203.411
	4	3	0	0	–
	4	0	3	0	–
	3	4	0	0	–
	3	0	4	0	–
DP8	8	0	0	0	2329.440
	8	0	0	1	2491.474
	7	1	0	0	2025.402
	7	0	1	0	2058.386
	6	2	0	0	–
	6	0	2	0	–
DP9	9	0	0	0	2617.529
	9	0	0	1	2779.519
	8	1	0	0	–
	8	0	1	0	2345.434
	8	1	0	1	2475.799
	8	0	1	1	2507.576
DP10	10	0	0	0	2905.564
	10	0	0	1	3067.662
	9	1	0	0	2602.470
	9	0	1	0	–
	8	2	0	1	–
	8	0	2	1	2523.489
DP11	11	0	0	0	3193.713
	11	0	0	1	3355.615
	10	1	0	0	–
	10	0	1	0	–
DP12	12	0	0	0	3481.673
	12	0	0	1	–
	11	1	0	0	–
	11	0	1	0	–

*Rhizophora* species are traditionally used for the treatment of pain [14], but no scientific support corroborates this action, especially for *R. mangle*. There are already works evidencing the positive therapeutic effect of *R. mangle* in the treatment of gastro intestinal diseases [15], which cause a high sensation of pain [16]. Banerjee et al. reported a significant reduction in pain severity and frequency of painful episodes, in patients who underwent alternative treatment with extracts rich in grape PAs, reported a significant reduction in the use of narcotic analgesics. The mechanism of action by which tannins act in the pain treatment is still poorly explained, but it is a reality [17]. In order to evaluate the effect of AERM in the treatment of pain, we performed experimental tests with formalin and tail-flick assays.

**Fig. 3:**
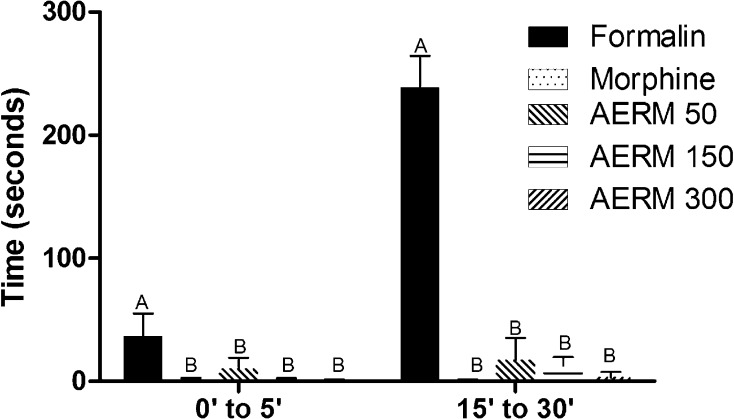
Analgesic effect of AERM (50, 150 and 300 μg·kg^−1^) on Formalin test. Results are expressed as mean ± SEM (n = 6), *p* < 0.0001

Treatment of animals with AERM at doses of 50, 150 and 300 µg kg^−1^ did not induce any sign of intercurrence in the animals. This test showed that the doses do not interfere in an animal’s behavior. The formalin test was performed to contribute to the tail-flick, whilst the former is better known to evaluate pain threshold or reflex through acute cutaneous stimulation, formalin injection induces a state that better approximates to clinical conditions, therefore evaluating tonic pain. Thus, in the first phase of the test, formalin predominantly evokes activity in C fibers, substance P and bradykinin, whilst histamine, serotonin, prostaglandins (PGs), and bradykinin are involved in the second phase [18]. Therefore, the formalin test allows the evaluation of analgesic effect in peripheral inflammatory processes, that is evoked especially in the second phase of the test. The stimulus provided by subcutaneous injection of formalin-induced a behavioral response in animals treated with formic acid, in contrast to animals treated with morphine and AERM compounds at different concentrations, which had almost no behavioral response to formalin injection in both phases of the formalin test. Therefore, the formalin test showed that there is no difference between the analgesic effect of morphine and bark compounds in the lowest concentration tested, even in a test that provides a long-lasting pain stimulus such as the formalin test. Despite the dose–response tendency presented, there were no differences between the extract’s concentrations tested in relation to the biological activity (Fig. 3; Table 3).

**Table 3:** Analgesic effect by formalin test of AERM (50, 150 and 300 µg kg^−1^)

Time (min)	Formalin	Morphine	AERM 50	AERM 150	AERM 300
0′–5′	36.54 ± 18.52^A^	1.08 ± 1.52^B^	10.10 ± 8.95^B^	1.05 ± 1.47^B^	0.50 ± 0.99^B^
15′–30′	238.80 ± 25.61^A^	0.51 ± 0.95^B^	17.03 ± 18.42^B^	12.77 ± 6.77^B^	3.23 ± 4.46^B^

Significant difference was marked with different letters in the same row (two-way ANOVA, mean and standard deviation, Bonferroni post hoc test, p < 0.01, F = 229.7)

**Fig. 4:**
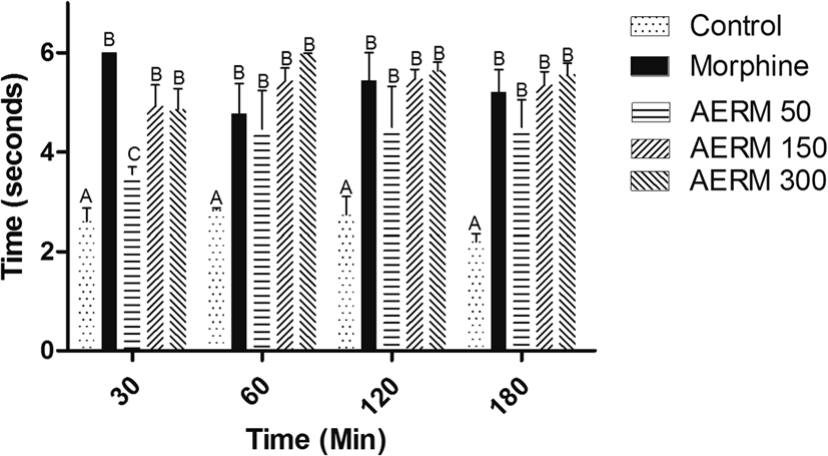
Analgesic effect of AERM (50, 150 and 300 μg·kg^−1^) on Tail-flick test. Results are expressed as mean ± SEM (n = 6), *p* < 0.0001

Tests with more phasic stimuli, such as the tail-flick tests, provide an evaluation of antinociceptive effect in acute pain state. In this scenario, the response to thermal stimuli was evaluated using the tail-flick test. As it was shown in Fig. 4 and Table 4, both the effect of morphine as well as the bark compounds in the lowest concentration, were substantially greater than in control (saline-treated animals). This result indicates that the AERM compounds had a spinal effect similar to morphine, though the exact mechanism is still unknown. The reason behind this morphine-like effect presented by the AERM may be due to the action of CAs, as it was shown previously that these substances act like blockers for sodium channels in neuronal cells [19]. Hence, this morphine-like effect observed for AERM has a significant clinical importance, as it could be used as a potential substitute for the morphine. Considering all the morphine’s adverse effects (sedation, nausea, and pruritus) [15], the use of the AERM could improve the treatment of pain, especially chronically.

**Table 4:** Analgesic effect by tail-flick test of AERM (50, 150 and 300 µg kg^−1^)

Time (min)	Control	Morphine	AERM 50	AERM 150	AERM 300
30	2.60 ± 0.46^A^	6.00 ± 0.00^B^	3.55 ± 0.21^C^	4.91 ± 0.88^B^	4.85 ± 0.96^B^
60	2.83 ± 0.058^A^	4.77 ± 1.07^B^	4.47 ± 1.33^B^	5.42 ± 0.60^B^	5.97 ± 0.04^B^
120	2.73 ± 0.63^A^	5.43 ± 0.98^B^	4.50 ± 1.41^B^	5.46 ± 0.40^B^	5.63 ± 0.43^B^
180	2.20 ± 0.26^A^	5.20 ± 0.80^B^	4.50 ± 0.95^B^	5.35 ± 0.60^B^	5.55 ± 0.58^B^

Significant difference was marked with different letters in the same row (two-way ANOVA, mean and standard deviation, Bonferroni post hoc test, p < 0.01, F = 46.27)

**Fig. 5:**
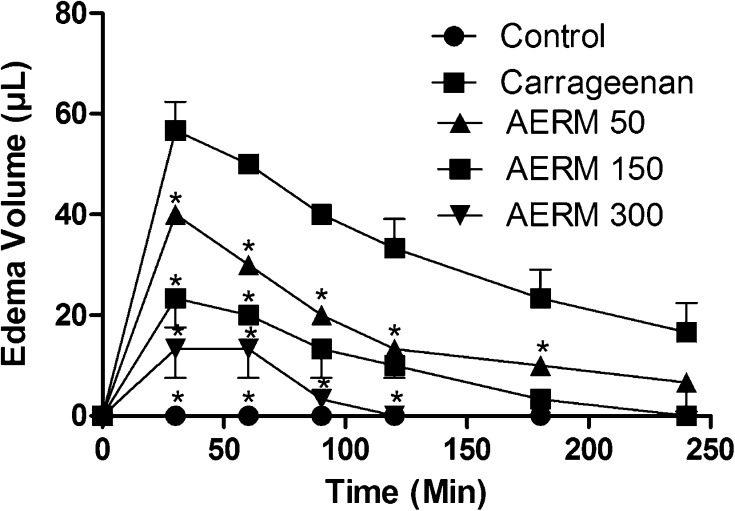
Monitoration of paw edema induced by carrageenan. The analysis showed an inhibition when treatments were injected with AERM. Two-way ANOVA with Bonferroni as a *post test* (*p* < 0.01). Statistical differences relative to carrageenan (filled squares) are marked with *

**Fig. 6:**
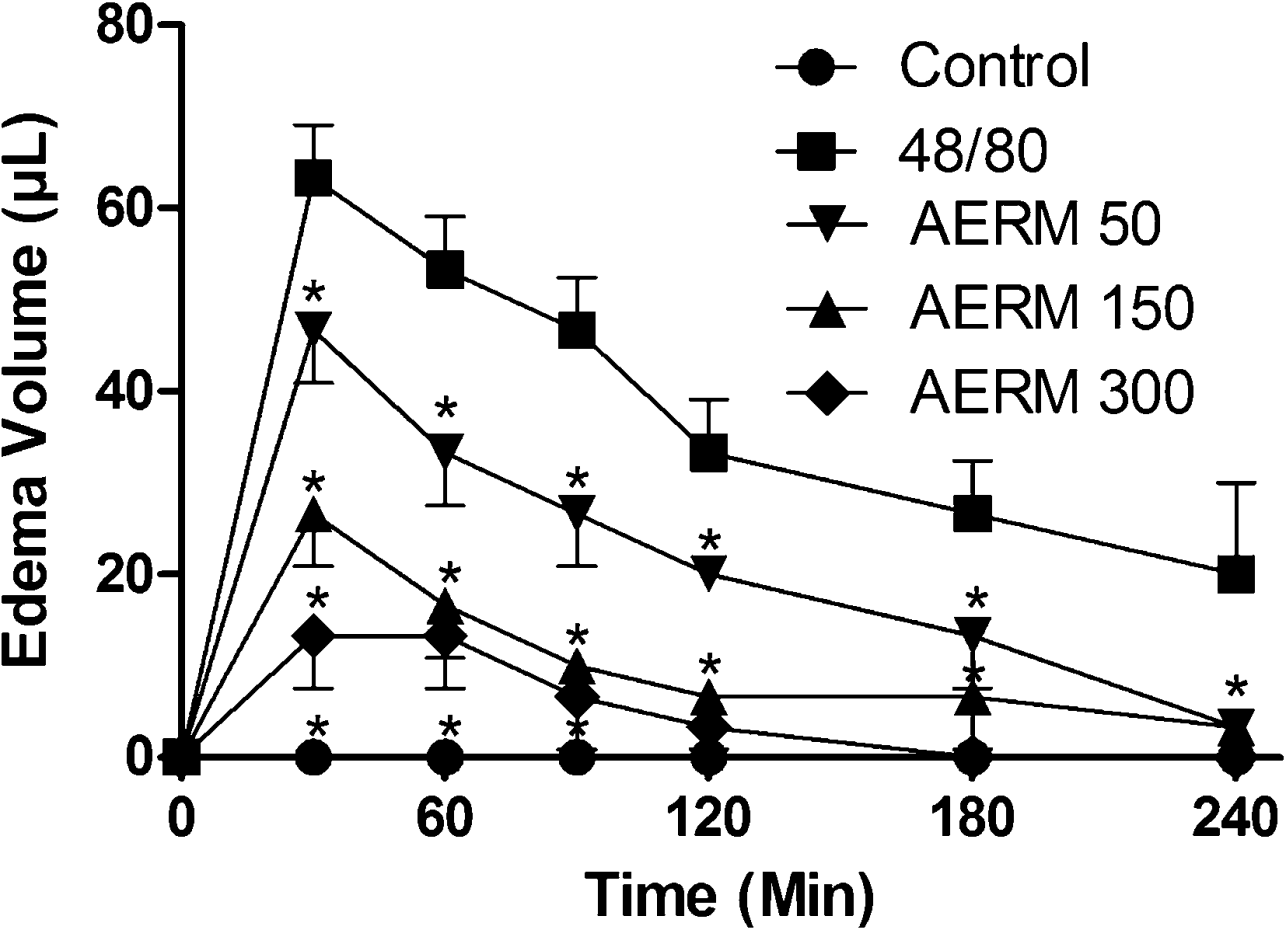
Monitoration of paw edema induced by 48/80. The analysis showed an inhibition when treatments were injected with AERM. Two-way ANOVA with Bonferroni as a *post test* (*p* < 0.001). Statistical differences relative to the compound 48/80 (filled squares) are marked with *

Pain may originate through an inflammatory process, several mediators such as cytokines, bradykinins and PGs are responsible for pain [20]. Our research group evidenced the anti-inflammatory activity of the ethyl acetate and butanol fractions of the AERM [21]. A scientific basis underpinned by the compounds of *R. mangle* may be helpful to explain its antinociceptive and anti-inflammatory effects. Pharmacological test of paw edema induced by carrageenan showed an anti-inflammatory potential, since there was the statistical difference (two-way analysis of variance, ANOVA with Bonferroni *post test*, F = 61.46, *p* < 0.01, Df = 5) (Fig. 5). Also, paw edema induced by compound 48/80 also demonstrate a large inhibition when AERM was injected (two-way ANOVA with Bonferroni *post test*, F = 43.00, *p* < 0.01, Df = 5) (Fig. 6).

The acute inflammatory response is characterized by an increase in vascular permeability and cellular infiltration leading to edema formation, because of extravasation fluid and proteins and accumulation of leukocytes at the inflammatory site [22]. Carrageenan-induced paw edema is a widely-used test to determine the anti-inflammatory activity and induced cyclo-oxygenase-2 (COX-2) maximal expression 1 h from carrageenan local injection. However, compound 48/80, like other basic secretagogues, causes mast cell degranulation via direct activation of Gi proteins (sensitive to Pertussis toxin), releasing oxygen reactive species (ROS). Degranulation of mast cells results in the release of stored autacoids, such as histamine, as well as the de novo synthesis of arachidonic acid (AA), cytokines and chemokines. Upon mast cell degranulation, AA is released and metabolized through COX or 5-lipoxygenase (5-LO) pathways to yield, respectively, PGs and thromboxane, or leukotrienes [23, 24]. So, phenolic compounds as found in *R. mangle* could be actin by controlling the mast degranulation as ROS scavenger and might inhibit specifically enzymes from the inflammatory process as COX-2 [25].

Furthermore, *Pinus pinaster* is also rich in polyphenolic compounds, such as afzelechins and galocatechins, same as found in *R. mangle*, and has been shown to have similar analgesic and anti-inflammatory effects as *R. mangle* [26]. *Pinus pinaster* cause inhibition of cyclo-oxygenase (COX-1 and COX-2) activity in serum samples of human volunteers. COXs enzymes are the major targets for the treatment of inflammation, fever and pain, due to its function, that is synthesis of mediators, such as PGs, prostacyclin, and thromboxane. Which suggests, that the COX blockage may also play a role in the analgesic and anti-inflammatory effect of *R. mangle* due to polyphenolic compounds activity.

Several anti-inflammatory drugs on market are known, however, they reveal side effects such as liver and kidney commitment [27]. Other species by genus *Rhizophora* have anti-inflammatory activity [28]. In *R. mangle*, inhibition of PLA2 and COX-2, enzymes related to inflammation process, demonstrated by in vitro tests [14], which reiterates the results revealed in this study. Kanagaratnam et al. [29] reported that CAs may act as an anti-inflammatory because they reveal antibacterial activity; which probably supports the results found in this work. Another mechanism capable of generating pain can originate in response to tissue damage or physiological stress, including oxidative stress [30]. The condensed tannins present in *R. mangle* have excellent free radical sequestration [21], thus, the antioxidant capacity of this extract can aid in the maintenance of pain and inflammation.

## Conclusions

This research showed the potential of UPLC coupled to ion trap tandem MS and MALDI-TOF analysis for the rapid and sensitive detection of natural phenolic polymers in the acetone extract from the barks of *R. mangle.* This technique allowed detecting the presence of a series of anthocyanidins in less than 5 min of analysis. MALDI-TOF MS allowed characterizing all the polymeric substances present in the extract, revealing the presence of a series of condensed tannins based on CAs, gallocatechins and afzelechins. Besides, these series were accompanied by their respective glucosilated derivatives. The identification of these compounds has provided relevant data for fully characterize the secondary metabolites present in these complex vegetal extracts. The chemical characterization of the extract also helped to understand the results observed in the formalin, tail-flick and paw edema assays using experimental models, which demonstrated the antinociceptive and anti-inflammatory effects of the AERM.

## Experimental Section

### Plant Material

The barks of *R. mangle* L. (Rhizophoraceae) were collected during August 2015 from the estuary of the ecological station of Juréia-Itatins (Peruíbe, São Paulo, Brazil, 24°25′40″S–47°05′20″ W). The collecting the material had prior authorization from the Brazilian authorities (IBAMA/MMA: 52497-1). The taxonomic identification of the plant was carried out by Prof. Paulo Sampaio. The voucher specimens (n° 11459) has been deposited at the Herbarium HUSC of the Santa Cecilia University (Santos, São Paulo, Brazil).

### Preparation of Plant Extract (AERM)

Fresh barks of *R. mangle* were washed, shade dried, powdered in a knife mill and sieved through a #60 mesh sieve. The powder (50 g) was extracted with 0.5 L acetone:water (70% v/v) and macerated for 7 days at room temperature (24 °C), protected from light. The macerate was filtered through Whatman No. 1 filter paper and concentrated in a rotating flash evaporator at a temperature not exceeding 35 °C. The extract was lyophilized and stored in amber glass flasks, stored in a freezer (− 40 °C) and resuspended with distilled water for use in pharmacological experiments. The percent yield of the lyophilized powder was 7% of the dry plant material.

### Phytochemical Evaluation

#### LC–ESI–MS/MS Analysis

MS analyses were carried out in a Thermo Finnigan (San Jose, CA, USA) LCQ mass spectrometer equipped with an electro-spray ionization source, ion-trap analyzer and Xcalibur software for data processing. For LC–MS analysis: column Phenomenex, 300 μL min^−1^, followed by chromatographic using MeOH and 0.002% formic acid in water was added using a linear gradient from 0 to 100% MeOH over 5 min. The mass spectrometer was operated in the negative ion mode under the following conditions: flow 5 μL min^−1^; the capillary temperature 270 °C; 80 arbitrary units of nitrogen; gas assist with 5 arbitrary units. All solvents used in these analyzes have a mass grade, purchased from Sigma.

#### MALDI-TOF

For the analysis using MALDI-TOF, 1.0 mg of the powdered vegetable material was diluted in 500 μL of MeOH containing 2,5-dihydroxybenzoic acid (DHB – 125 nm) and sodium iodide (1 nM) with the addition of 1% trifluoroacetic acid (v/v), purchased from Sigma. This solution was placed for 1 min by vortexing. After this, 1 μL of the solution suspension has been deposited on MALDI plates, made of stainless steel and allocated to air dry prior to analysis. The analysis of MALDI-MS was carried out a Voyager DE-RP coupled to the spectrometer flight time mass (TOF), equipped with a nitrogen laser (λ = 337 nm, pulse width 3rd). The intensity of the laser beam was experimentally attenuated slightly above the threshold level for forming ions from the tannin components. The ions generated by laser desorption were introduced in the flight tube with a 20 kV accelerating voltage in positive linear mode. The delay time is 100 ns. All mass spectra were acquired by averaging 100 individual laser shots [31].

### Experimental Analysis

#### Animals

Ethical approval for the animal studies was obtained from the Animal Use Committee of the São Paulo State University, reference number: 019 CEUA/CLP, UNESP, São Paulo State University/Coastal Campus of São Vicente, on August 2016. The experiments were performed with a minimum number of animals that provides a robust analysis. Healthy adult female Wistar rats weighing 25–30 g at 8–12 weeks (n = 6) The experiments were performed in independent duplicates and were obtained from Campinas University (Campinas, São Paulo, Brazil). The animals were kept in standard environmental conditions: temperature (23 ± 2 °C), light control (12 h light cycle) and provide water and food ad libitum (standard rodent diet), except during the experimental period. All animals were acclimated for a period of 5 days before the start of the experimental assays.

#### Formalin Assay

Formalin test was done according to the modified method of [32]. Each animal was placed inside a box (30 × 30 × 30 cm, length × width × height) after formalin injection in right foot plantar area (1% v/v). Thus, Swiss female mice fasted for a period of 8 h and divided into five groups (n = 6), Control (NaCl 0.9%), Morphine (50 µg kg^−1^, i.p.) and AERM treated with *R. mangle* extracts (50, 150 and 300 µg kg^−1^). After 1 h of each treatment, animals were given an intraplantar formalin injection (20 µL; 2%). Two responses were analyzed: acute-phase (0–5 min) and later-phase (15–30 min) [33, 34], it is considered the zero time point when formalin was immediately injected.

#### Tail-Flick Assay

Tail-flick test was evaluated of central analgesic activity [35]. The latency period is used as a determination index of nociception. Data collected from mice with latency lower than 2 s were not used, and 6 s was the maximum latency tolerance. Animals were divided into five groups (n = 6), Control (saline solution), Morphine (50 µg kg^−1^, i.p.) and AERM (50, 150 and 300 µg kg^−1^, i.p.), an analgesymeter (Insight EFF 300L), with the tail docked in apparatus with the light thermal source was used. The latency of tail withdrawal response was determined 0, 30, 60, 120 e 180 min after the AERM administration, inject peritoneally.

#### Paw Edema Test Induced by Carrageenan and 48/80 Compounds

AERM was injected peritoneally (50, 150 and 300 µg mL^−1^), 10 min after carrageenan (1.5%) (n = 6), and 48/80 (2%) (n = 6) in Swiss mice. For controls, we used saline (NaCl 0.9%). The monitoring of edema volume was using plethysmometer for 4 h or until diminution of inflammation reaches 20% of the original.

### Statistical Analysis

All data were expressed as a mean ± standard error of the mean. Statistical analyses were performed by using the Statistical Package for Social Science version 15.0 (SPSS) and GraphPad Prism 5.0. The statistical significance was determined using a two-way ANOVA, followed by Bonferroni post hoc test, a level of 5% (*p* < 0.05) was considered to be statistically significant.
